# Proteomic analysis of human placental syncytiotrophoblast microvesicles in preeclampsia

**DOI:** 10.1186/1559-0275-11-40

**Published:** 2014-11-19

**Authors:** Sonia Baig, Narasimhan Kothandaraman, Jayapal Manikandan, Li Rong, Kim Huey EE, Jeffrey Hill, Chin Wee Lai, Wan Yu Tan, Felicia Yeoh, Anita Kale, Lin Lin Su, Arijit Biswas, Sheila Vasoo, Mahesh Choolani

**Affiliations:** Division of Maternal-Fetal Medicine, Department of Obstetrics & Gynaecology, Yong Loo Lin School of Medicine, National University of Singapore & National University Health System, 10 Medical Drive, Singapore, 119260 Singapore; Division of Rheumatology, Department of Medicine, Yong Loo Lin School of Medicine, National University of Singapore & National University Health System, 10 Medical Drive, Singapore, 119260 Singapore; Experimental Therapeutic Center, Agency for Science, Technology and Research, 31 Biopolis Way, Singapore, 138669 Singapore

**Keywords:** Placental microvesicles, Pregnancy, Preeclampsia, Proteome

## Abstract

**Background:**

Placental syncytiotrophoblast microvesicles (STBM) are shed into the maternal circulation during normal pregnancy. STBM circulate in significantly increased amounts in preeclampsia (PE) and are considered to be among contributors to the exaggerated proinflammatory, procoagulant state of PE. However, protein composition of STBM in normal pregnancy and PE remains unknown. We therefore sought to determine the protein components of STBM and whether STBM protein expressions differ in preeclamptic and normal pregnancies.

Patients with PE (n = 3) and normal pregnant controls (n = 6) were recruited. STBM were prepared from placental explant culture supernatant. STBM proteins were analyzed by a combination of 1D Gel-LC-MS/MS. Protein expressions levels were quantified using spectral counts and validated by immunohistochemistry.

**Results:**

Over 400 proteins were identified in the STBM samples. Among these, 25 proteins were found to be differentially expressed in preeclampsia compared to healthy pregnant controls, including integrins, annexins and histones.

**Conclusion:**

STBM proteins include those that are implicated in immune response, coagulation, oxidative stress, apoptosis as well as lipid metabolism pathways. Differential protein expressions of STBM suggest their pathophysiological relevance in PE.

**Electronic supplementary material:**

The online version of this article (doi:10.1186/1559-0275-11-40) contains supplementary material, which is available to authorized users.

## Background

Microvesicles and exosomes are small (<1 μm) membrane-bound structures generated by cells after activation or apoptosis. They are important vehicles of intercellular communication carrying membrane, cytosolic and nuclear proteins, lipids, messenger RNAs and microRNAs [1–4]. Human placental syncytiotrophoblast microvesicles (STBM) are shed into the maternal circulation in normal pregnancy and are significantly increased in preeclampsia (PE) [5]. STBM are proinflammatory [6–8], procoagulant [9], and anti-angiogenic [10, 11] in normal pregnancy. Some of these characteristics are known to be exaggerated in (PE) [9, 10]; however the basis of pathogenicity of preeclamptic STBM is yet to be elucidated.

Proteomes of microvesicles derived from platelets, plasma, malignant lymphocytes, endothelial cells, dendritic cells, mast cells, and intestinal epithelial cells have been published [12–18]. Broadly, microvesicle proteins are either ubiquitous or specific to cells of origin. Ubiquitous proteins are most likely involved in microvesicle biogenesis. Examples include tetraspanins (CD9, CD63, CD81 and CD82), heat shock proteins (HSP70, HSP90), cytoskeletal proteins (tubulin, actin, actin binding proteins), metabolic enzymes, membrane transport and fusion proteins (annexins and RAB proteins), signal transduction proteins (protein kinases, 14-3-3, G proteins), integrins and MHC class I and II molecules [19]. Examples of cell-specific proteins include MHC class II in exosomes from all cells expressing MHC class II, CD 86 from DC-derived exosomes (a co-stimulatory molecule for T cells), T-cell receptors for T-cell derived exosomes, and immunoglobulin family members (CD54 on B cells, P-selectin on platelets). In contrast to exosomes, microvesicles released by apoptotic cells do contain nuclear and organeller proteins, DNA, messenger and microRNA. Both exosomes and microvesicles may provide unconventional routes of protein secretion [20].

Multiple pathophysiologic factors are implicated in adverse pregnancy outcomes such as PE, including maternal immune maladaptation to the feto-placental unit, excessive fetal trophoblast apoptosis, and increased shedding of trophoblast debris. These result in increased systemic inflammatory response, haemostatic activation, endothelial dysfunction, and metabolic changes [21]. Defective trophoblast invasion resulting in abnormal uteroplacental perfusion and oxidative stress in PE may result in qualitative and subsequent functional changes in STBM. Quantitative changes in serum, placental and decidual lipid and protein oxidation products and anti-oxidant concentrations are significantly associated with PE [22]. Proteomic analyses of normal and PE placentas [23], syncytiotrophoblasts [24], cytotrophoblasts [25–27], deciduas [28], and plasmas [29, 30] have been previously reported. While the role of placenta in PE is a vibrant area of research, the important question of why STBM from preeclamptic placenta may have pro-inflammatory and hypertensive effects on the maternal systemic vasculature remain unanswered. In particular, proteins of placental STBM in normal and preeclamptic pregnancy have not been determined yet.

We hypothesized that STBM proteins are differentially expressed in PE compared to normal pregnancies. Our objectives were to determine protein and peptide components of STBM that can incite pathogenic responses in pregnancy complications and explore whether these STBM protein expressions differ in pathologic (i.e., PE) and normal pregnancies. As opposed to earlier studies which employed 2D PAGE and MALDI TOF/TOF [23, 31], and 2D DIGE [29], we used 1D Gel-LC-MS/MS approach to improve sensitivity [30]. Also, the current study aimed to investigate protein composition of placental microvesicles in health and disease which has not been reported to date.

## Results

### Subject characteristics

We studied 3 pregnant women complicated with PE and 6 healthy normal gestational age-matched pregnant women. The clinical characteristics of women and maternal and neonatal outcomes are summarized in Table 1. The patients underwent caesarean section or vaginal delivery at gestational age >34 weeks with live births.

**Table 1:** **Maternal and fetal outcomes**

	PE (n = 3)	Normal controls (n = 6)
maternal age (y)	31.[2.16]^a^	36.33[5.28]
Gestational age (w)	36.09[0.95]	36.38[1.56]
SBP (mm Hg)	173.25[31]***	116.25[15.31]
DBP (mm Hg)	109.5[13.2]***	76.5[5.58]
Proteinuria (g/24/h)	1.14[0.57]	-
Infant birth wt (g)	2658[792.02]	2722.5[434.79]
Placental wt (g)	508.33[79.74]	526.80[94.17]

^a^Results are mean [SD], comprarisons using Student’s test (patient group, ie, PE versus normal pregnant controls), P<0.0001***.

### STBM characterization

STBM characterization data are summarized in Additional file 1: Figure S1. H/E and immunofluorescent (PLAP) staining of placental villous explants demonstrated the syncytiotrophoblast layer (Additional file 1: Figure S1- A & B). Scanning electron microscopy demonstrated expected size of STBM (Additional file 1: Figure S1- C). Nanoparticle tracking analysis [32] showed STBM size distribution to be between 30–300 nm with a peak around 100 nm (Additional file 1: Figure S1- D).

### Identification of STBM proteins by 1D Gel-LC-MS/MS

**Figure 1:**
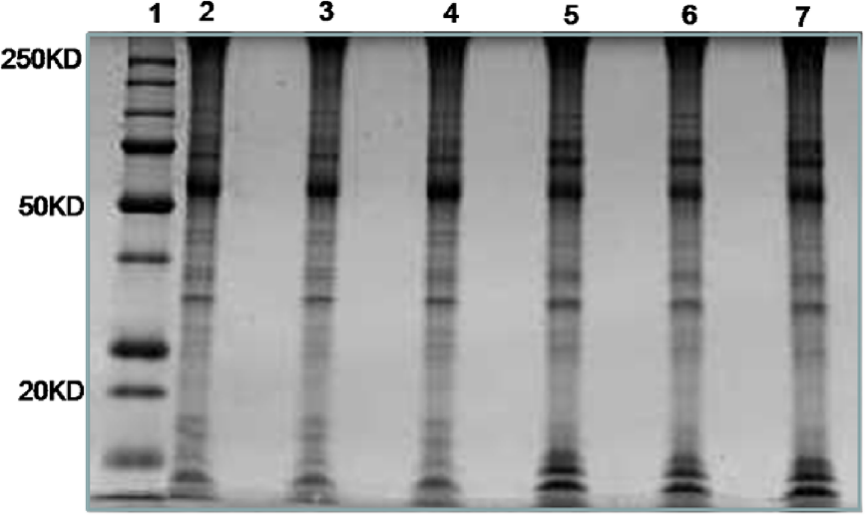
**Representative 1-D SDS PAGE gel images of protein extracts from STBM.** Lane 1: molecular weight marker. Lanes 2, 3 & 4: Normal STBM. Lanes 5, 6 & 7: PE (preeclampsia) STBM. Loading Volume: 20 uL per lane (~50 ug of proteins).

Representative 1D gels are shown in Figure 1. After in-gel digestion, the tryptic peptides of each gel slice were analyzed by LC-MS/MS. Over 400 STBM proteins were identified (Additional file 2: Table S1). Proteins were designated as hits only when there were at least 2 unique peptides matches and Protein Identification Probability value of >95%. Spectral counts were used to quantify expression level of proteins in the whole lane.

### Identification of differentially expressed STBM proteins in adverse pregnancies and healthy controls

We identified 25 STBM proteins to be differentially expressed between preeclamptic and normal pregnancies, listed in Table 2; notable among them are annexins, integrins, histones, heat shock proteins, complement regulatory proteins, cytoskeletal proteins, and various enzymes. Histones, integrins and CD59 glycoprotein were downregulated while the remaining were upregulated in preeclampsia. The Cellular components of STBM proteins are summarized in Additional file 3: Figure S2.

**Table 2:** **Differential protein expressions in STBM isolated from patients with PE (n = 3) versus that from pregnant controls (n = 6)**

^a^Protein name	Symbol	Accession number	Molecular weight	^b^Mean PE	^c^SEM PE	Mean NP	SEM NP	p-value
isoform 1 of Annex in A2	ANXA2	IPI004553 15	39 kDa	-	-	66.32	16.12	-
isoform 2 of Annex in A2	ANXA2	IPI00418169	40 kDa	94.32	13.51	-	-	-
annex in A4	ANXA4	IPI00793199	36 kDa	109.78	24.56	47.36	9.72	0.0227
Histone H 14	HST1HiE	IPI00217467	22 kDa	-	-	23.84	4.47	-
Histone 1.5	HST1H1B	IPI00217468	23 kDa	-	-	6.56	169	-
Isoform 1 of Intergrin alpha-V	ITGAV	IPI00027505	116 kDa	-	-	25.60	4.02	-
Isoform Beta-1A of Intergrin beta-1	ITGB 1	IPI00217563	88 kDa	4.24	4.24	19.15	3.43	0.0353
Glyceraldehyde-3-phospahte dehydrogenase	GAPDH	IPI00219018	36 kDa	51.76	22.95	9.48	3.18	0.0312
Heat schock protein beta-1	HSPB	IPI00025512	23 kDa	13.54	6.24	2.85	0.76	0.0398
Isoform 1 of Heat shock cognate 71 kDa protein	HSPA8	IPI00003865	71 kDa	10.31	3.99	1.97	0.67	0.0202
14-3-3 protein epsillon	YWHAE	IPI00000816	29 kDa	11.18	3.17	-	-	-
Actin, cytoplasmic 1	ACTB	IPI00021439	42 kDa	35.21	13.53	-	-	-
ADP-ribosylation factor 1	ARF 1	IPI00215914	21 kDa	6.72	2.60	-	-	-
Alpha-actinin-1	ACTN 1	IPI00013508	103 kDa	21.58	1.97	-	-	-
CD59 glycoprotein	CD59	IPI00011302	14 kDa	-	-	9.61	2.11	-
EF-hand domain-containing protein D1	EFHD1	IPI00031091	27 kDa	5.87	3.48	-	-	-
Ezrin	EZR	IPI00843975	69 kDa	40.30	15.01	1.17	0.98	0.0056
HSPA5 protein	HSPA 5	IPI00003362	72 k Da	10.80	3.96	-	-	-
Isoform 1 of Brain acid soluble protein 1	BASP1	IPI00299024	23 kDa	15.57	4.32	6.29	1.54	0.0373
Isoform alpha-enolase of Alpha-eno lase	EN01	IPI00465248	47 kDa	12.22	6.22	1.96	0.69	0.0446
Isoform M2 of Pyruvate Kinase Isozymes M*v*M2	PKM	IPI00479186	58 kDa	7.45	4.15	-	-	-
Transketo lase	TKT	IPI00643920	69 kDa	9.67	4.49	1.33	0.95	0.0374
Tubullin alpha-1C cahin	TUBA 1C	IPI00218343	50 kDa	10.24	4.47	0.33	0.33	0.0125
Tubullin beta-2C cahin	TUBB4B	IPI00007752	50 kDa	5.98	3.10	-	-	-
^d^Tubullin, beta	TUBB	IPI00645452	48 kDa	6.13	3.89	-	-	-

^a^Only proteins with a fold change >2 or <0.5 and p-value <0.05 or expressed only in one group (either PE or NP) are listed.

^b^Numbers represent total spectral from all the peptides of a given protein.

^c^Results are mean and standard error of mean (SEM), comparisons using t test between patients with preciamsia (PE n = 3) versus that from normal pregnant conrols (NP, n = 6).

^d^For complete list of all STBM proteins see Additional file 4: Table S2 in Supplementary data.

### Validation of proteomics results by immunohistochemistry (IHC) analysis

IHC analysis of fixed and paraffin-embedded placental villous explants for validation of LC-MS/MS-based STBM-proteomics data confirmed the presence of selected STBM proteins in the syncytiotrophoblast layer at periphery while negative controls showed no signals (Additional file 4: Table S2, Additional file 5: Figure S3). Statistically non-significant trends towards downregulation of these proteins, namely, histones, annexins, and integrins, could be observed (Additional file 6: Figure S4).

### Functional and pathway analysis

**Figure 2:**
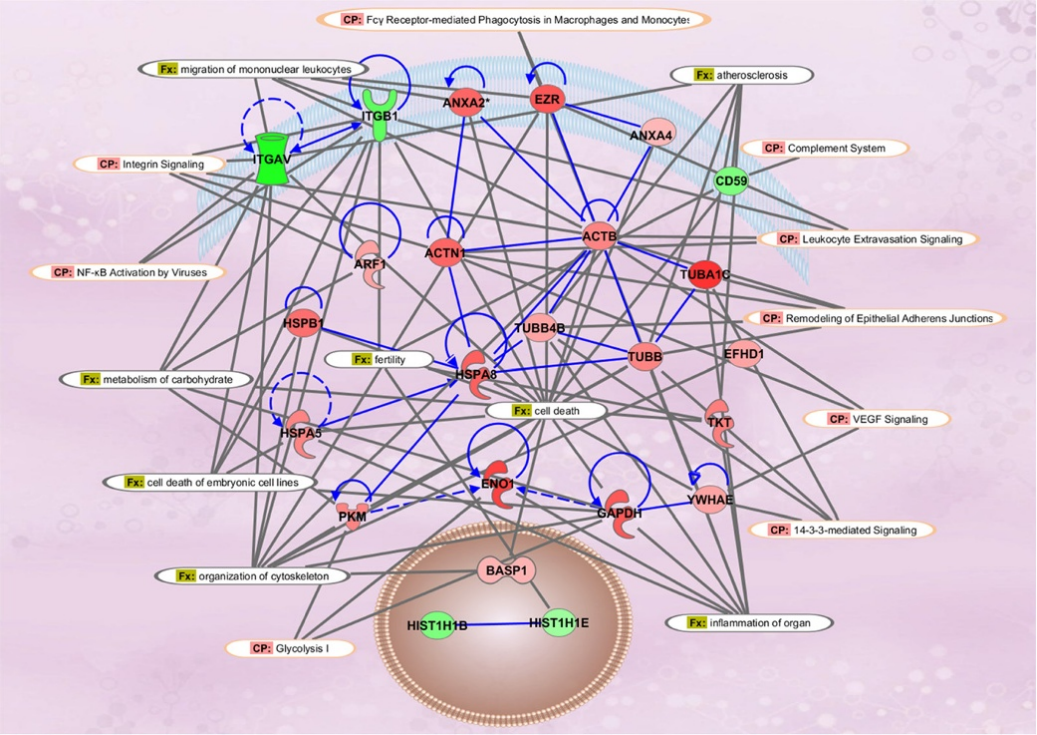
**Proposed biological network of differentially expressed proteins.** Using Ingenuity Pathways Analysis software (IPA), selected differentially expressed proteins were utilized to build a biological network based on previously reported interactions in the literature. The expression levels of proteins were overlaid on to the network. Molecules colored with red denote upregulation and green denotes downregulation. Finally, canonical pathways and functions were mapped on the existing network. CP: Canonical Pathway; FX: Functions.

Ingenuity Pathways Analysis (IPA) of statistically differentially expressed dataset containing 25 unique STBM proteins identified the significant biological functions and pathways. The major biological functions of STBM proteins include cell death and survival, cellular assembly and organization, immune response, lipid metabolism, and carbohydrate metabolism (Figure 2, Additional file 7: Table S3). The major canonical pathways include signaling associated with glycolysis, inter-cellular junctions, integrins, endothelial dysfunction (VEGF), immune response (NF-kB and complement system) and protein ubiquitination (Figure 2, Additional file 8: Table S4).

## Discussion

We sought to determine the protein composition of STBM using 1D gel-LC-MS/MS approach to gain an insight into the pathophysiologic changes of PE. To our knowledge this study is the first report of STBM proteome in PE in comparison to that of normal healthy pregnancies.

Twenty five proteins were found to be differentially expressed between STBM derived from women with PE compared to healthy controls, including annexins, integrins, histones, and heat shock proteins. The annexins are calcium dependent-phospholipid binding proteins involved in various functions including signal transduction, stress response, inflammation, coagulation, apoptosis and lipid metabolism. Annexin A2 has been proposed to function inside the cell in sorting of endosomes and outside the cell in anticoagulant reactions [33]. The upregulation of anti-apoptosis proteins in PE STBM, such as annexin A4 (2-fold change, p < 0.05) [34], can be explained by increased apoptotic activity of the placental syncytiotrophoblasts in PE which would trigger the generation of anti-apoptotic proteins in an attempt to counteract pro-inflammatory molecules such that homeostasis is achieved. Glyceraldehyde-3-phosphate dehydrogenase, a component of glycolytic pathway, has important functional relevance in anaerobic glycolysis in trophoblasts enriched in glycogen. Its increased expression in PE STBM (5-fold change, p < 0.05) could be associated with oxidative stress that exists in these adverse pregnancies [35, 36]. Reduced expression of complement regulatory protein such as CD59 could be observed, suggestive of a possible role of STBM in hemostatic activation, as well as regulation [37]. Collectively all these protein expressions possibly depict the syncytiotrophoblast response at the maternal-fetal interface to underlying pregnancy pathology in PE.

Integrin signaling has an important role in trophoblast invasion and vasculature remodeling [38–40]. Its down regulation in PE STBM may be associated with shallow trophoblast invasion and defective placental vasculature in PE. Ezrin is involved in cell surface structure adhesion, migration and organization [41, 42]. A trend towards increased expression of proteins such as 14-3-3 proteins, annexins and heat shock proteins may represent a protective response to tissue injury in pathologic pregnancies [43].

Major functions of histones include DNA binding, DNA repair and transcriptional regulation [44]. The unique finding of downregulation of histones in PE STBM, in this study (0.1 to 0.3-fold change, p < 0.05), may be suggestive of defective DNA repair, increase in damaged DNA and raised inflammatory response in adverse pregnancies.

Interestingly, we found that STBM proteins include endogenous danger molecules or alarmins which may render them intensely pro-inflammatory such as extracellular free actins, tubulins, and heat shock proteins [3]. This supports the currently emerging role of STBM as contributors to the pro-inflammatory state of PE.

Future biomarker discovery work will include validation of biomarker potentials of differentially expressed proteins in patients’ plasma. We acknowledge the limitations of using the label-free method and spectral counts in demonstrating the true representation of differential expression of low abundance proteins. Confirmation of the proteins found to be differentially expressed in this study needs to be carried out with isotope-encoded peptides corresponding to the tryptic peptides of the selected proteins. This will help determine absolute concentration of targeted proteins.

## Conclusions

The present study suggests that the biological pathways associated with PE may include immune response, coagulation, endothelial dysfunction as well as lipid metabolism. We have recently reported differential lipid expressions in STBM from the same cohort of patients [45]. While previous evidence for increased activation of hemostasis and endothelial dysfunction exists, the concept and proposed roles of immune-dysregulation and lipid metabolism in pathophysiology of adverse pregnancies are novel and yet to be established. Our findings are consistent with recent reports on PE pregnancy sera [29, 30], demonstrating pro-inflammatory and lipid-regulatory protein expressions, besides those involved in hemostasis and endothelial dysfunction. Targeting these novel pathways may offer newer strategies of management of adverse pregnancies.

## Methods

### Subjects and sample collection

The study subjects were patients with PE. PE was defined as increased blood pressure (≥140/90 mm Hg) that occurred after 20 wks of gestation in previously normotensive women accompanied with proteinuria of ≥0.3 g/day. The controls were gestational age-matched (within 2 gestational weeks) healthy subjects with no pregnancy complications. Placental samples were collected at the time of delivery and processed within 2 hours. All samples were collected from the National University Hospital affiliated to the National University of Singapore, Singapore. The research protocol was approved by the Domain Specific Review Board.

### 5.2 Placental microvesicle (STBM) preparation and characterization

Placental villous tissue (~2 gm) was cut into small pieces (2–4) mm^3^ and rinsed in PBS until blood less. Explants were cultured in 100 mm culture dishes in DMEM-F12 culture medium, supplemented with 1% antimycotic/antibiotics and 10% heat-inactivated fetal bovine serum, for generation of STBM at 37°C in 8% oxygen in the hypoxic incubator (Galaxy 48R CO_2_ incubator with O_2_ control 0.1 to 19% range). Prior to addition to the culture media, the heat-inactivated fetal bovine serum was centrifuged at 100 000 g for 60 min for removal of microvesicles in serum. At the end of 72 hours culture period, the supernatant was collected followed by differential centrifugation for isolation of STBM.

Culture supernatant was subjected to a three-step centrifugation at 4°C - 1000 g for 10 min (separation of whole cells), 10,000 g for 10 min (separation of large cellular organelles, such as mitochondria, golgi, ER, etc.) and finally, 100 000 g for 60 min (for collecting subcellular membrane microvesicles). The final pellet was collected, washed in sterile PBS and resuspended in 0.5 ml sterile PBS and stored at -20°C until use. The protein content in each STBM preparation was quantified with Bradford assay (Pierce, Rockford, IL). Typical STBM preparations yielded 0.2-0.4 mg/ml vesicles. STBM size and number in sample was determined by Nanoparticle Tracking Analysis (NTA) method (Dragovic et al. [32]). Finally STBM samples were subjected to evaluation scanning electron microscopy (SEM). Additional 48 hr culture of aliquots of explant culture supernatants was carried out prior to STBM isolation to confirm the absence of bacterial growth and endotoxin contamination.

### Protein separation, in-gel digestion and LC-MS/MS analysis

The STBM samples prepared from placental explant culture supernatant were pelleted down. STBM pellets were then resuspended in RIPA lysis buffer (Pierce, Rockford, IL), homogenized by vortexing and lysed on ice for 30 minutes. The sample was centrifuged at 14,000 g for 15 minutes at 4°C and the supernatant (lysate) was collected.

The protein lysate derived from the STBM was then subjected to precipitation overnight at -20°C after addition of five volumes of pre-cooled acetone. Protein precipitation was followed by reduction with 10 mM dithiothreitol (DTT) and alkylation with 20 mM iodoacetamide (IAA). The protein content in each STBM preparation was quantified with Bradford assay (Pierce, Rockford, IL). Proteins (~50 μg) were separated on a 10.5-14% Tris–HCl SDS-gel (BIO-RAD) and stained with PageBlue™ protein staining solution (Fermentas) containing Coomassie Brilliant Blue G-250 dye. 1D SDS-PAGE system (BIO-RAD) was used to separate the protein lysate derived from STBM.

Each lane in gel was finely cut into 13 slices, transferred into a 96 wells plate with hole and in-gel digestion was performed as follows. First, destaining was performed by the addition of 100 ul of 100 mM TEAB/EtOH (1:1, vol/vol) and incubated with occasional vortexing for 60 min at 56°C, followed by addition of 100 ul of neat acetonitrile and incubation at room temperature with occasional vortexing, until gel pieces became white and dried. Then acetonitrile was removed. Buffer containing trypsin (20 ng/ul) was added in an amount to cover the dry gel pieces (typically, 50 ul or more, depending on the volume of a gel matrix) and left in an ice bucket or a fridge about 15 min. After 15 min, it was checked if all solution was absorbed and more trypsin containing buffer was added, if necessary. After full rehydration the remaining trypsin buffer was spun out, then 10–20 ul of 10 mM TEAB buffer was added to cover the gel pieces and keep them wet during enzymatic cleavage. Plate with gel pieces was placed into an air circulation thermostat and samples were incubated overnight at 37°C. Plate was chilled to room temperature and 5 ul 5%formic acid (FA) was added to stop digestion. Solution was spun down into new 96 wells collection plate using a plate centrifuge followed by addition of 50 ul 1%FA twice and 50 ul of 1:2(vol/vol) 0.1% formic acid/acetonitrile to extract peptide from gel. 300 ul aliquots of the supernatant directly from the digest extracted from the gel pieces was used for the subsequent MS/MS analysis. Samples were freeze-dried and the dried protein film was then reconstituted in formic acid prior to analysis by mass spectrometry.

For LC MS/MS analysis, 10 ul of 0.1% (vol/vol) formic acid was added into each well to resuspend peptides. 5 ul sample was injected to Agilent 6538 chip LCMSMS system for analysis after separation by Agilent 1200 LC system, using a 1D nano -LC setup consisting of a capillary pump for loading the sample (operated at 4 uL/min) onto 1.0-cm trap column and a nano pump (operated at 300 nL/min) for the separation on the analytical column. 100% water with 0.1% formic acid was used as solvent A and 100% Acetonitrile with 0.1% formic acid was used as solvent B. The peptides were eluted from the precolumn using a gradient from 95% phase A (0.1% FA aqueous solution) to 45% phase B (0.1% FA, 100% acetonitrile) in 60 min at 300 nl/ min directly onto an analytical column (50-um inner diameter, 360-um outer diameter, ReproSil-Pur C18 5um). The instrument was operated in a data-dependent mode automatically switching between MS, MS2. Acquisition was set to automatically select and further fragment the fragment ion originating from the loss of phosphoric acid from the parent ions (standard pdMS2 settings).

### LC-MS/MS data processing and quantitative analysis

Generated mass spectral data were analyzed using Mascot sequence matching software (Matrix Science). General search parameters used were: Enzyme, Trypsin; Maximum of 2 missed cleavages; Fixed modifications, Carbamidomethyl (C); Variable modifications, Oxidation (M), Deamidated (NQ), Phospho (STY), Acetyl (K); Peptide tolerance range ±100 ppm; and MS/MS tolerance range, ±0.6 Da. Data import filters used were Mascot Distiller (Matrix Science). Analyzed database-search results (.dat) from Mascot were uploaded onto Scaffold interface. Result files were categorized and named according to sample type or nature. Multidimensional protein identification technology (MuDPIT) experiment and condensation of loaded data were selected. Proteins were designated as hits only when there were at least 2 unique peptides matches and Protein Identification Probability value of >95%.

The quantitation of protein expressions by spectral counts for each identified proteins (defined as total spectral counts for all the peptides of a given protein) was carried out using Scaffold program (Proteome Software, version 4). Only proteins with spectral counts ≥5 and a ratio of spectral counts between two groups (patients and normal controls) of >2 or <0.5 were tested further by *t*-test and validation by immunohistochemistry.

### Immunohistochemistry (IHC)

Villous explants were fixed and paraffin-embedded. Tissue microarrays were prepared. IHC was performed according to manufacturer’s instructions (Leica Microsystems). Briefly, after deparaffinization and heat-induced epitope retrieval, endogenous peroxidase blocking was performed. Slides were incubated with primary antibodies for 45 minutes. Antibodies used are listed in Additional file 2: Table S1. After washing, the sections were exposed to anti-rabbit poly-HRP-IgG in 10% animal serum for 10 minutes, followed by DAB reagents for 3 minutes. DAB reaction was stopped, and counterstaining with hematoxylin was performed for 5 minutes. After washing, sections were dehydrated and finally mounted in synthetic mounting media (Electron Microscopy Services, Hatfield, USA). Controls included omission of primary antibodies. After IHC, tissue microarray slides were scanned, analyzed and scored (Leica Microsystems slide scanner and Slidepath image analysis software).

### Functional and pathway analysis

To define biological networks, interaction and functional analysis among the differentially expressed proteins, pathway analyses were performed using Ingenuity Pathways Analysis software (IPA) (Ingenuity Systems, Redwood City, CA). Statistically differentially expressed dataset containing 25 unique proteins and their corresponding IPI identifier, p-value and fold change values were uploaded into the IPA. The significance of the connection between the expression data and the canonical pathway were calculated by ratio and/or Fisher’s exact.

### Statistical analysis

Analysis of statistical significance of differences in protein spectral counts between patient group and normal healthy pregnant group were sought using Student’s two-tailed unpaired *t*-test. Analyzes were performed using GraphPad Prism 5 software. Results were considered to be statistically significant if *p* < 0.05.

### Supplementary information

Supplementary Information includes four supplementary tables and four supplementary figures and can be found with this article online.

## Electronic supplementary material

Additional file 1: Figure S1: Representative images of STBM basic characterization. A. H/E staining of placental villous explants showing deep blue syncytiotrophoblast (STB) layer at periphery (X20); B. Immunofluorescence (IF) image of placental villous explants demonstrating the bright green layer of STB stained for Placental Alkaline Phosphatase, PLAP (X40); C. Representative scanning electron microscopy image of STBM by (X10.000); D. Representative light scatter of STBM by nanoparticle tracking analysis (NTA), showing STBM size to be between 30-300 nm with a peak around 100 nm. (PPT 2 MB)

Additional file 2: Table S1: Antibodies used for Immunohistochemistry. (XLS 14 KB)

Additional file 3: Figure S2: Cellular components of STBM proteins. The placental membrane microvesicles, ie, STBM carry membrane, cytoskeletal and regulatory proteins. (PPT 94 KB)

Additional file 4: Table S2: List of STBM proteins. (XLS 74 KB)

Additional file 5: Figure S3: Representative Immunohistochemistry images of formalin-fixed paraffin-embedded placental villous explants from women with preeclampsia (PE) and normal healthy pregnancy. Proteins are localized in the syncytiotrophoblasts. Bars represent 100 μm (original magnification X200). (PPT 1 MB)

Additional file 6: Figure S4: Summary of Immunohistochemical validation of PE STBM protein expressions. Comparison of mean staining intensity units of individual proteins in STBM from PE patients (n=3) and normal pregnant women (n=6). (PPT 164 KB)

Additional file 7: Table S3: Functional analysis of the differentially regulated proteins. (XLS 26 KB)

Additional file 8: Table S4: Canonical pathways predicted by Ingenuity Pathway Analysis. (XLS 25 KB)

## Abbreviations

STBM: Syncytiotrophoblast microvesicle
PE: Preeclampsia
PLAP: Placental alkaline phosphatase
NTA: Nanoparticle tracking analysis.

## References

[1] Gupta AK, Holzgreve W, Hahn S (2008). Decrease in lipid levels of syncytiotrophoblast micro- particles reduced their potential to inhibit endothelial cell proliferation. Arch Gynecol Obstet.

[2] Cocucci E, Racchetti G, Meldolesi J (2009). Shedding microvesicles: artefacts no more. Trends Cell Biol.

[3] Redman CW, Tannetta DS, Dragovic RA, Gardiner C, Southcombe JH, Collett GP, Sargent IL (2012). Review: does size matter? Placental debris and the pathophysiology of pre-eclampsia. Placenta.

[4] Rajakumar A, Cerdeira AS, Rana S, Zsengeller Z, Edmunds L, Jeyabalan A, Hubel CA, Stillman IE, Parikh SM, Karumanchi SA (2012). Transcriptionally active syncytial aggregates in the maternal circulation may contribute to circulating soluble fms-like tyrosine kinase 1 in preeclampsia. Hypertension.

[5] Knight M, Redman CW, Linton EA, Sargent IL (1998). Shedding of syncytiotrophoblast microvilli into the maternal circulation in pre-eclamptic pregnancies. Br J Obstet Gynaecol.

[6] Germain SJ, Sacks GP, Sooranna SR, Sargent IL, Redman CW (2007). Systemic inflammatory priming in normal pregnancy and preeclampsia: the role of circulating syncytiotrophoblast microparticles. J Immunol.

[7] Messerli M, May K, Hansson SR, Schneider H, Holzgreve W, Hahn S, Rusterholz C (2009). Feto-maternal interactions in pregnancies: placental microparticles activate peripheral blood monocytes. Placenta.

[8] Southcombe J, Tannetta D, Redman C, Sargent I (2011). The immunomodulatory role of syncytiotrophoblast microvesicles. PLoS One.

[9] Gardiner C, Tannetta DS, Simms CA, Harrison P, Redman CW, Sargent IL (2011). Syncytiotrophoblast microvesicles released from pre-eclampsia placentae exhibit increased tissue factor activity. PLoS One.

[10] Smarason AK, Sargent IL, Starkey PM, Redman CW (1993). The effect of placental syncytiotrophoblast microvillous membranes from normal and pre-eclamptic women on the growth of endothelial cells in vitro. Br J Obstet Gynaecol.

[11] Gupta AK, Rusterholz C, Holzgreve W, Hahn S (2005). Syncytiotrophoblast micro-particles do not induce apoptosis in peripheral T lymphocytes, but differ in their activity depending on the mode of preparation. J Reprod Immunol.

[12] Garcia BA, Smalley DM, Cho H, Shabanowitz J, Ley K, Hunt DF (2005). The platelet microparticle proteome. J Proteome Res.

[13] Jin M, Drwal G, Bourgeois T, Saltz J, Wu HM (2005). Distinct proteome features of plasma microparticles. Proteomics.

[14] Miguet L, Pacaud K, Felden C, Hugel B, Martinez MC, Freyssinet JM, Herbrecht R, Potier N, van Dorsselaer A, Mauvieux L (2006). Proteomic analysis of malignant lymphocyte membrane microparticles using double ionization coverage optimization. Proteomics.

[15] Wubbolts R, Leckie RS, Veenhuizen PT, Schwarzmann G, Mobius W, Hoernschemeyer J, Slot JW, Geuze HJ, Stoorvogel W (2003). Proteomic and biochemical analyses of human B cell-derived exosomes. Potential implications for their function and multivesicular body formation. J Biol Chem.

[16] Thery C, Regnault A, Garin J, Wolfers J, Zitvogel L, Ricciardi-Castagnoli P, Raposo G, Amigorena S (1999). Molecular characterization of dendritic cell-derived exosomes. Selective accumulation of the heat shock protein hsc73. J Cell Biol.

[17] Thery C, Boussac M, Veron P, Ricciardi-Castagnoli P, Raposo G, Garin J, Amigorena S (2001). Proteomic analysis of dendritic cell-derived exosomes: a secreted subcellular compartment distinct from apoptotic vesicles. J Immunol.

[18] van Niel G, Raposo G, Candalh C, Boussac M, Hershberg R, Cerf-Bensussan N, Heyman M (2001). Intestinal epithelial cells secrete exosome-like vesicles. Gastroenterology.

[19] Thery C, Zitvogel L, Amigorena S (2002). Exosomes: composition, biogenesis and function. Nat Rev Immunol.

[20] Nickel W (2005). Unconventional secretory routes: direct protein export across the plasma membrane of mammalian cells. Traffic.

[21] Roberts JM, Lain KY (2002). Recent Insights into the pathogenesis of pre-eclampsia. Placenta.

[22] Serdar Z, Gur E, Colakoethullary M, Develioethlu O, Sarandol E (2003). Lipid and protein oxidation and antioxidant function in women with mild and severe preeclampsia. Arch Gynecol Obstet.

[23] Gharesi-Fard B, Zolghadri J, Kamali-Sarvestani E (2009). Proteome differences of placenta between pre-eclampsia and normal pregnancy. Placenta.

[24] Okamura K, Powell JE, Lee AC, Stevens VC (1981). Characterization of solubilized microvillous membrane proteins and glycoproteins from human placental syncytiotrophoblast. Placenta.

[25] Hu R, Jin H, Zhou S, Yang P, Li X (2007). Proteomic analysis of hypoxia-induced responses in the syncytialization of human placental cell line BeWo. Placenta.

[26] Johnstone ED, Sawicki G, Guilbert L, Winkler-Lowen B, Cadete VJ, Morrish DW (2011). Differential proteomic analysis of highly purified placental cytotrophoblasts in pre-eclampsia demonstrates a state of increased oxidative stress and reduced cytotrophoblast antioxidant defense. Proteomics.

[27] Epiney M, Ribaux P, Arboit P, Irion O, Cohen O (2012). Comparative analysis of secreted proteins from normal and preeclamptic trophoblastic cells using proteomic approaches. J Proteomics.

[28] Menkhorst EM, Lane N, Winship AL, Li P, Yap J, Meehan K, Rainczuk A, Stephens A, Dimitriadis E (2012). Decidual secreted factors alter invasive trophoblast membrane and secreted proteins implying a role for decidual cell regulation of placentation. PLoS One.

[29] Blumenstein M, McMaster MT, Black MA, Wu S, Prakash R, Cooney J, McCowan LM, Cooper GJ, North RA (2009). A proteomic approach identifies early pregnancy biomarkers for preeclampsia: novel linkages between a predisposition to preeclampsia and cardiovascular disease. Proteomics.

[30] Liu C, Zhang N, Yu H, Chen Y, Liang Y, Deng H, Zhang Z (2010). Proteomic analysis of human serum for finding pathogenic factors and potential biomarkers in preeclampsia. Placenta.

[31] Shin JK, Baek JC, Kang MY, Park JK, Lee SA, Lee JH, Choi WS, Paik WY (2011). Proteomic analysis reveals an elevated expression of heat shock protein 27 in preeclamptic placentas. Gynecol Obstet Invest.

[32] Dragovic RA, Gardiner C, Brooks AS, Tannetta DS, Ferguson DJ, Hole P, Carr B, Redman CW, Harris AL, Dobson PJ, Harrison P, Sargent IL (2011). Sizing and phenotyping of cellular vesicles using Nanoparticle Tracking Analysis. Nanomedicine.

[33] Takahashi S, Reddy SV, Chirgwin JM, Devlin R, Haipek C, Anderson J, Roodman GD (1994). Cloning and identification of annexin II as an autocrine/paracrine factor that increases osteoclast formation and bone resorption. J Biol Chem.

[34] El Kebir D, Jozsef L, Filep JG (2008). Opposing regulation of neutrophil apoptosis through the formyl peptide receptor-like 1/lipoxin A4 receptor: implications for resolution of inflammation. J Leukoc Biol.

[35] Tarze A, Deniaud A, Le Bras M, Maillier E, Molle D, Larochette N, Zamzami N, Jan G, Kroemer G, Brenner C (2007). GAPDH, a novel regulator of the pro-apoptotic mitochondrial membrane permeabilization. Oncogene.

[36] Zala D, Hinckelmann MV, Yu H, da Cunha MM L, Liot G, Cordelières FP, Marco S, Saudou F (2013). Vesicular glycolysis provides on-board energy for fast axonal transport. Cell.

[37] Huang Y, Qiao F, Abagyan R, Hazard S, Tomlinson S (2006). Defining the CD59-C9 binding interaction. J Biol Chem.

[38] Murai JT, Muzykanskiy E, Taylor RN (1997). Maternal and fetal modulators of lipid metabolism correlate with the development of preeclampsia. Metabolism.

[39] Redfern CH, Degtyarev MY, Kwa AT, Salomonis N, Cotte N, Nanevicz T, Fidelman N, Desai K, Vranizan K, Lee EK, Coward P, Shah N, Warrington JA, Fishman GI, Bernstein D, Baker AJ, Conklin BR (2000). Conditional expression of a Gi-coupled receptor causes ventricular conduction delay and a lethal cardiomyopathy. Proc Natl Acad Sci U S A.

[40] Huppertz B, Berghold VM, Kawaguchi R, Gauster M (2012). A variety of opportunities for immune interactions during trophoblast development and invasion. Am J Reprod Immunol.

[41] Granés F, Urena JM, Rocamora N, Vilaró S (2000). Ezrin links syndecan-2 to the cytoskeleton. J Cell Sci.

[42] Yun CH, Lamprecht G, Forster DV, Sidor A (1998). NHE3 kinase A regulatory protein E3KARP binds the epithelial brush border Na+/H + exchanger NHE3 and the cytoskeletal protein ezrin. J Biol Chem.

[43] Benoit C, Zavecz J, Wang Y (2007). Vasoreactivity of chorionic plate arteries in response to vasoconstrictors produced by preeclamptic placentas. Placenta.

[44] Liang D, Burkhart SL, Singh RK, Kabbaj MH, Gunjan A (2012). Histone dosage regulates DNA damage sensitivity in a checkpoint-independent manner by the homologous recombination pathway. Nucleic Acids Res.

[45] Baig S, Lim JY, Fernandis AZ, Wenk MR, Kale A, Su LL, Biswas A, Vasoo S, Shui G, Choolani M (2013). Lipidomic analysis of human placental Syncytiotrophoblast microvesicles in adverse pregnancy outcomes. Placenta.

