# Supplementary figures and images for: Proteomic analysis of human placental syncytiotrophoblast microvesicles in preeclampsia
Source: Clin Proteomics. 2014 Nov 19;11(1):40. doi: 10.1186/1559-0275-11-40 (PMC4247627; doi:10.1186/1559-0275-11-40)

## Slide 1
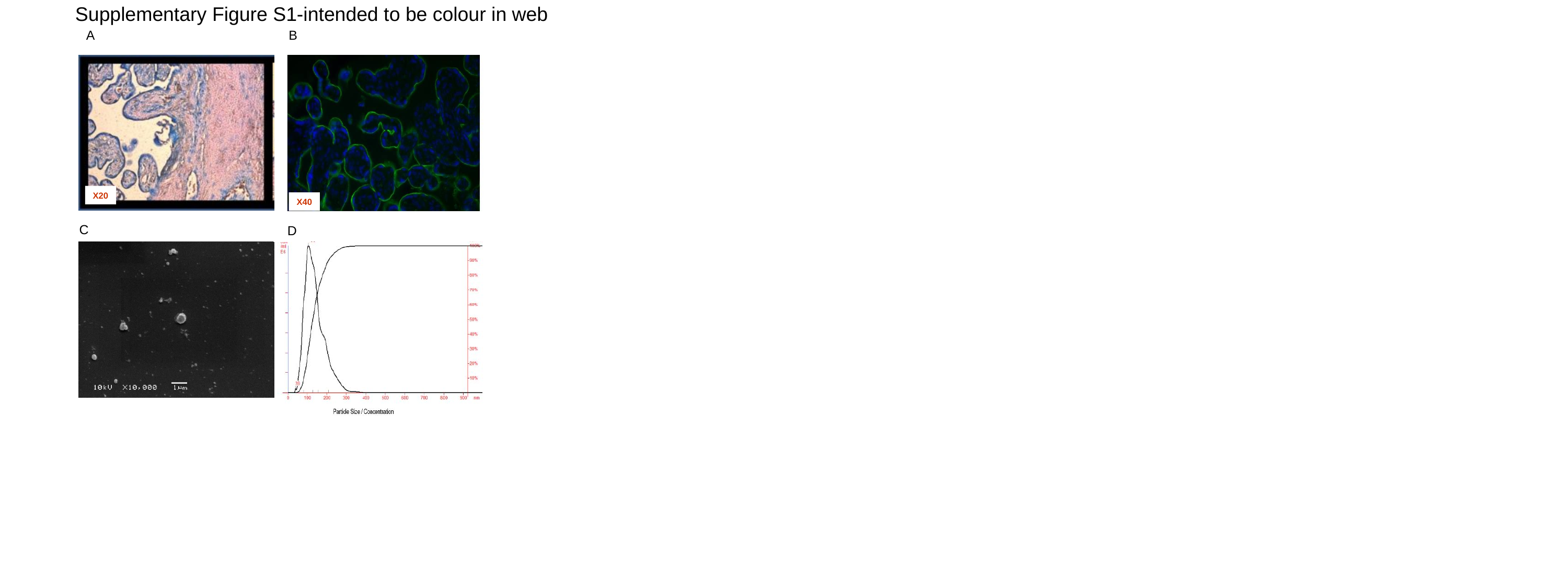

Supplementary Figure S1-intended to be colour in web
A
B
X20
X40
C
D

Supplement: Supplementary file 1 — Additional file 1: Figure S1: Representative images of STBM basic characterization. A. H/E staining of placental villous explants showing deep blue syncytiotrophoblast (STB) layer at periphery (X20); B. Immunofluorescence (IF) image of placental villous explants demonstrating the bright green layer of STB stained for Placental Alkaline Phosphatase, PLAP (X40); C. Representative scanning electron microscopy image of STBM by (X10.000); D. Representative light scatter of STBM by nanoparticle tracking analysis (NTA), showing STBM size to be between 30-300 nm with a peak around 100 nm. (PPT 2 MB) [file 12014_2014_82_MOESM1_ESM.ppt]

## Slide 1
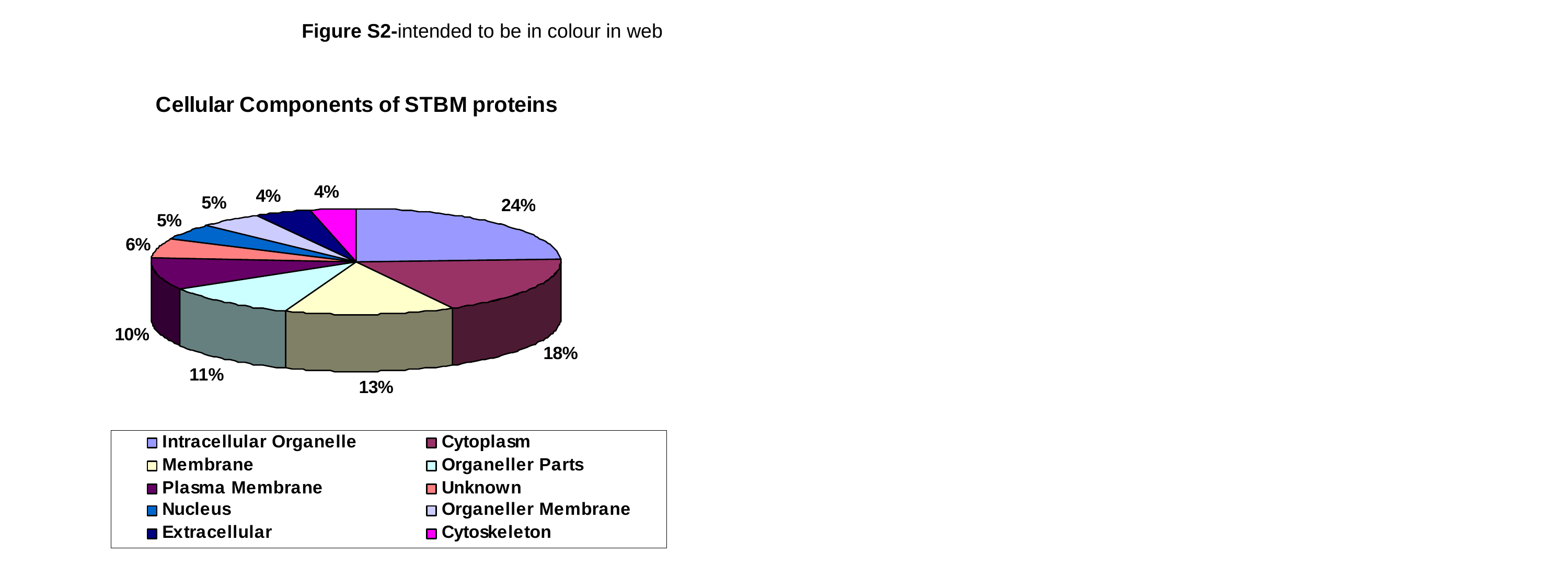

Figure S2-intended to be in colour in web

Supplement: Supplementary file 3 — Additional file 3: Figure S2: Cellular components of STBM proteins. The placental membrane microvesicles, ie, STBM carry membrane, cytoskeletal and regulatory proteins. (PPT 94 KB) [file 12014_2014_82_MOESM3_ESM.ppt]

## Slide 1
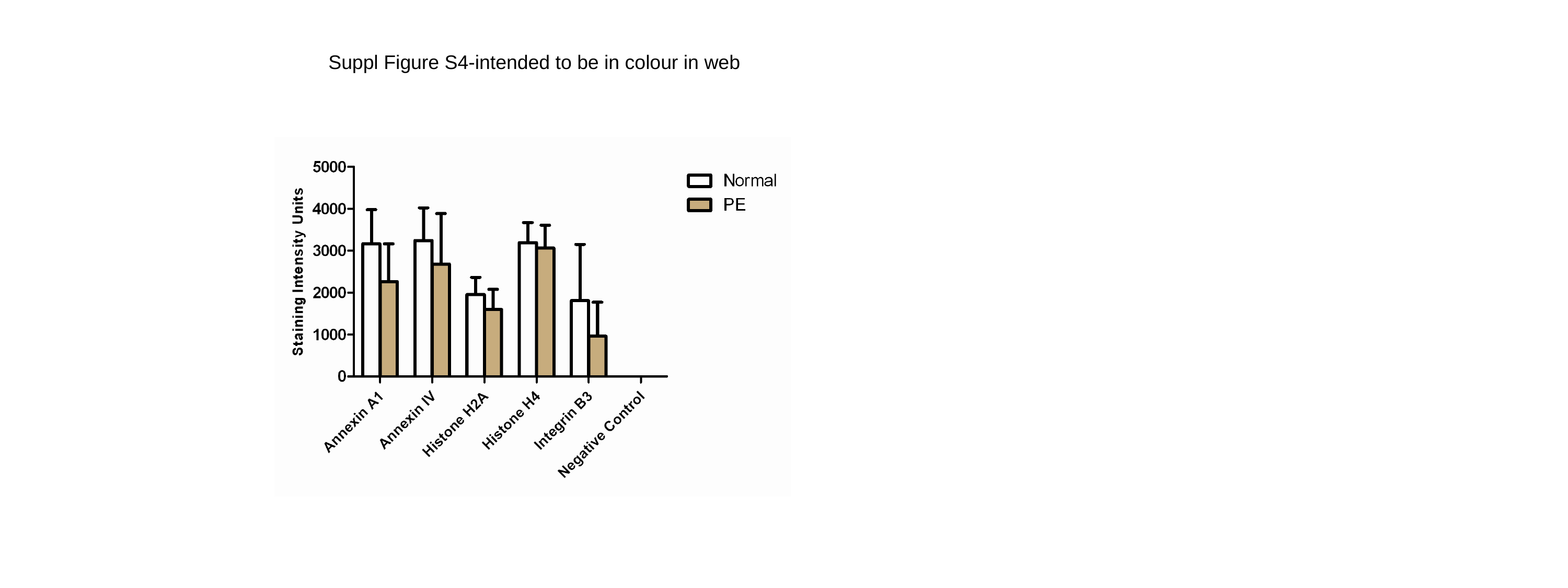

Suppl Figure S4-intended to be in colour in web

Supplement: Supplementary file 6 — Additional file 6: Figure S4: Summary of Immunohistochemical validation of PE STBM protein expressions. Comparison of mean staining intensity units of individual proteins in STBM from PE patients (n=3) and normal pregnant women (n=6). (PPT 164 KB) [file 12014_2014_82_MOESM6_ESM.ppt]
