# Supplementary material for: Proteomic analysis of human placental syncytiotrophoblast microvesicles in preeclampsia
Source: Clin Proteomics. 2014 Nov 19;11(1):40. doi: 10.1186/1559-0275-11-40 (PMC4247627; doi:10.1186/1559-0275-11-40)
Supplement: Supplementary file 5 — Additional file 5: Figure S3: Representative Immunohistochemistry images of formalin-fixed paraffin-embedded placental villous explants from women with preeclampsia (PE) and normal healthy pregnancy. Proteins are localized in the syncytiotrophoblasts. Bars represent 100 μm (original magnification X200). (PPT 1 MB) [file 12014_2014_82_MOESM5_ESM.ppt]

## Slide 1
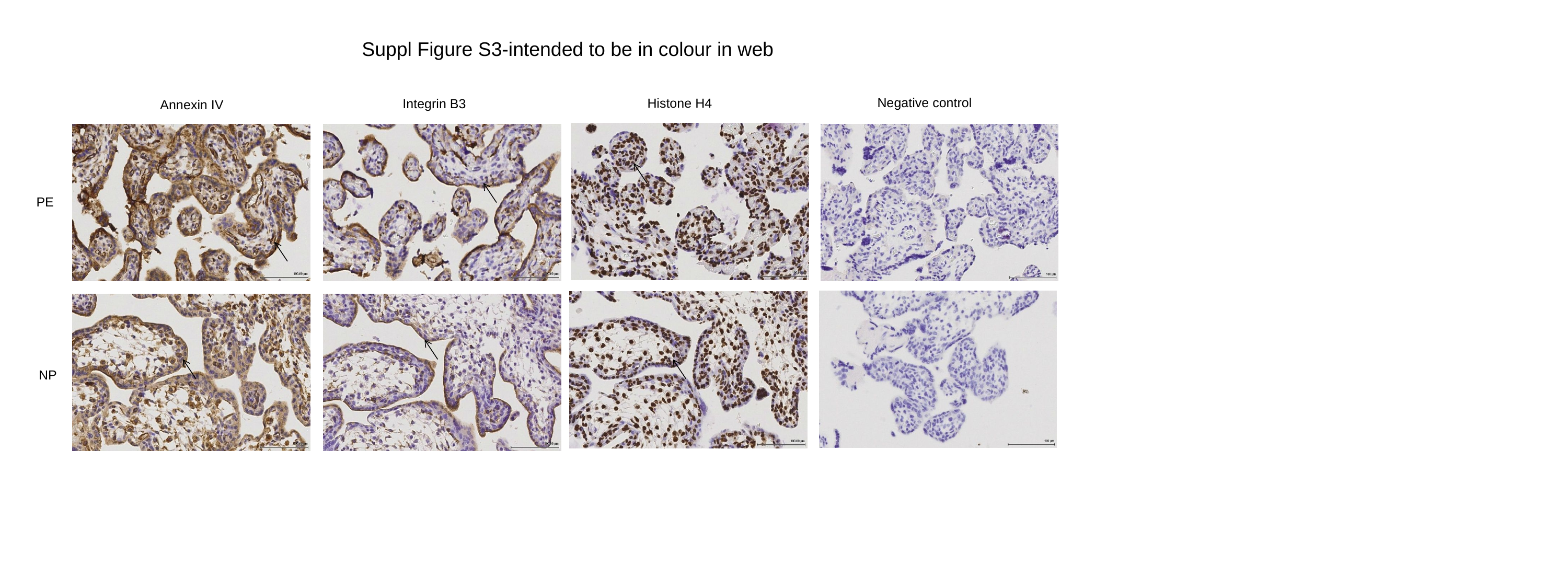

Suppl Figure S3-intended to be in colour in web
Negative control
Histone H4
Integrin B3
Annexin IV
PE
NP
